# Prevalence and clinical significance of *BRCA1/2* germline and somatic mutations in Taiwanese patients with ovarian cancer

**DOI:** 10.18632/oncotarget.13456

**Published:** 2016-11-19

**Authors:** Angel Chao, Ting-Chang Chang, Nina Lapke, Shih-Ming Jung, Peter Chi, Chien-Hung Chen, Lan-Yan Yang, Cheng-Tao Lin, Huei-Jean Huang, Hung-Hsueh Chou, Jui-Der Liou, Shu-Jen Chen, Tzu-Hao Wang, Chyong-Huey Lai

**Affiliations:** ^1^ Department of Obstetrics and Gynecology, Chang Gung Memorial Hospital, Linkou Medical Center, Taoyuan, Taiwan; ^2^ Gynecologic Cancer Research Center, Chang Gung Memorial Hospital, Taoyuan, Taiwan; ^3^ ACTGenomics, Co. Ltd., Taiwan; ^4^ Department of Pathology, Chang Gung Memorial Hospital, Linkou Medical Center, Taoyuan, Taiwan; ^5^ Institute of Biochemical Sciences, College of Life Science, National Taiwan University, Taiwan; ^6^ Clinical Trial Center, Chang Gung Memorial Hospital, Taoyuan, Taiwan; ^7^ School of Traditional Chinese Medicine, College of Medicine, Chang Gung University, Taoyuan, Taiwan

**Keywords:** BRCA1/2, germline mutations, somatic mutations, ovarian cancer

## Abstract

Germline and somatic *BRCA1/2* mutations define a subset of patients with ovarian cancer who may benefit from treatment with poly (ADP-ribose) polymerase inhibitors. Unfortunately, data on the frequency of *BRCA1/2* germline mutations in Taiwanese patients with ovarian cancer are scarce, with the prevalence of somatic mutations being unknown. We aim to investigate the occurrence of *BRCA1/2* mutations in 99 Taiwanese patients with ovarian cancer which included serous (n = 46), endometrioid (n = 24), and clear cell (n = 29) carcinomas. *BRCA1/2* mutations were identified using next-generation sequencing of formalin-fixed paraffin-embedded tumor samples. Pathogenic variants (*BRCA1*: n = 7; *BRCA2*: n = 6) were detected in 12.1% (12/99) of the study patients. Somatic and germline *BRCA1/2* mutation rates in serous ovarian cancer are 4/46 (8.7%) and 8/46 (17%), respectively. All of the pathogenic *BRCA1/2* mutations were identified in serous carcinoma samples (12/46; 26.1%). One-third (4/12) of the deleterious *BRCA1/2* mutations occurred in tumor tissues only (somatic mutations). All of them coexisted with loss of heterozygosity, resulting in biallelic *BRCA* inactivation. Five novel pathogenic mutations were identified, including four somatic variants (*BRCA1* p.S242fs, *BRCA1* p.F989fs, *BRCA1* p.G1738fs, and *BRCA2* p.D1451fs) and a germline variant (*BRCA2* p.E260fs). We also detected additional six novel mutations (three in BRCA1 and three in BRCA2) with pathogenic potentials. We conclude that *BRCA1/2* mutations are common in Taiwanese patients with serous ovarian carcinoma and similar to mutation rates in other ethnic groups. The analysis of *BRCA1/2* somatic mutations is crucial for guiding therapeutic decisions in ovarian cancer.

## INTRODUCTION

Ovarian cancer is the eighth most common cause of cancer death in Taiwanese women [1]. Approximately 238,700 new cases are diagnosed each year worldwide, which are responsible for 151,900 deaths [2]. The 5-year survival rates of ovarian cancer are stage-dependent and range between 27% and 92% [3]. Common adverse prognostic factors include advanced stages and disease recurrence [4].

Mutations of the *BRCA1/2* genes occur in approximately 20% of high-grade ovarian serous carcinoma [5] and are associated with better survival outcomes [6–10]. Inactivating *BRCA1/2* mutations portend an increased risk of malignant transformation because of their ability to impair homologous recombination-dependent DNA repair [11]. Conversely, inactivation of homologous recombination renders *BRCA1/2* mutant tumors sensitive to platinum [6, 8, 10, 12] and poly (ADP-ribose) polymerase (PARP) inhibitors (PARPi) [13]. Unfortunately, the identification of patients who could benefit from PARPi remains challenging.

The United States (US) Food and Drug Administration (FDA) approved the use of the PARPi olaparib when *BRCA1/2* germline mutations are present [14, 15]. However, the US FDA did not approve olaparib for patients who carry somatic *BRCA1/2* mutations only (approximately one third of all *BRCA1/2* mutant patients) [5, 6]. In contrast, the European Medicines Agency (EMA) approved olaparib for ovarian cancer patients who have either germline or somatic *BRCA1/2* mutations [13, 16]. Besides such regulatory discrepancies, not all patients with pathogenic *BRCA* mutations successfully respond to PARPi. A potential explanation lies in the fact that *BRCA* mutations require a loss of heterozygosity (LOH) to cause biallelic *BRCA* inactivation [17]. Although the loss of a single *BRCA* allele is sufficient *per se* to induce genomic instability and drive malignant transformation [18, 19], cells with monoallelic *BRCA* inactivation might not be sensitive to PARPi [20]. Unfortunately, the occurrence of biallelic inactivation of *BRCA1/2* has not been specifically analyzed in most of the available studies. In addition, few data on somatic and germline *BRCA1/2* mutations have been published in patients with Asian descent, and for Taiwanese patients, even the prevalence of germline mutations is only insufficiently investigated [21]. Notably, clear cell carcinomas are underrepresented in most studies because of their low incidence in North America and Europe (1−12% of all cases) compared to Asia (13−25%) [22–24]. *BRCA1/2* mutations have been reported in clear cell carcinomas [8, 25, 26] and endometrioid tumors [6, 8, 27–29], but their clinical significance requires further scrutiny.

Although Sanger sequencing is traditionally used for identifying *BRCA* mutations in clinical samples [30], next generation sequencing (NGS) has recently allowed obtaining a complete coverage of all exonic regions. This is essential since *BRCA* mutations differ among patients of different ethnicity [31]. Somatic *BRCA1/2* mutations can be successfully identified by NGS in formalin-fixed paraffin-embedded (FFPE) samples, but biallelic inactivation has not been thus far investigated [29].

Using NGS (covering all of the *BRCA1/2* exons as well as the exon-intron junctions) of FFPE specimens obtained from 99 Taiwanese patients with ovarian cancer, we analyzed 1) germline and somatic *BRCA1/2* mutations and 2) the occurrence of biallelic *BRCA1/2* inactivation.

## RESULTS

### Analytic workflow

We included 99 patients with ovarian cancer who were unselected for their family history of malignancies. The histological subtypes included serous (n = 46), endometrioid (n = 24), and clear cell (n = 29) carcinomas. Supplementary Figure S1 shows the workflow for the identification of BRCA1/2 variants identified in the current study.

### Detection of *BRCA1* and *BRCA2* variants by NGS and Sanger sequencing

NGS was used for *BRCA1/2* sequencing of FFPE tumor samples (average sequencing depth: >5700×; mean uniformity: 91.1%). Forty-four nonsynonymous and splice variants were identified in 36 patients. Of them, 13 were pathogenic variants (Table 1), 23 VUS (Supplementary Table S1), and eight benign variants. NGS-identified variants with an allele frequency >10% were confirmed by Sanger sequencing of tumor samples. To distinguish between germline and somatic mutations, Sanger sequencing was also performed for normal tissues (for all pathogenic variants; 13/13 and for all VUS with available control samples; 22/23). In addition to Sanger sequencing, NGS was performed in normal control samples when a sufficient amount of DNA was available (for pathogenic variants; 10/13 and for VUS; 18/23). A 100% concordance rate between the results of NGS and Sanger sequencing in tumor samples was observed.

**Table 1:** *BRCA* variant description and clinical characteristics for patients with pathogenic BRCA1/2 mutations

ID	Germline (G)/ Somatic (S)	Gene	Change for nucleotides (nt) and amino acids (aa)#		Variant classification (ARUP/ BIC/ BRCA Share/ ClinVar/ LOVD)	Type§	Age (y)	FIGO stage/grade	FH¶
			nt	aa					
Recurrent variants (n = 2)									
1	G	BRCA2	c.5164_5165 delAG	p.S1722fs	path/ path/ NA/ path/ path	Ser	50	IV/ 3	No
2	G	BRCA2	c.5164_5165 delAG	p.S1722fs	path/ path/ NA/ path/ path	Ser	57	I/ 3	No
Unique variants (n = 11)									
3	G	BRCA1	c.2188G>T	p.E730*	path/ path/ NA/ path/ NA	Ser	53	IV/ 3	NA
4	G	BRCA1	c.2387C>T	p.T796I	NA/ VUS/ NA/ VUS/ path	Ser	42	III/ 3	No
5	G	BRCA1	c.5332+1G>A	p.Œ_splice	path/ VUS/ path/ path/ path	Ser	48	III/ 3	No
6	G	BRCA1	c.3858_3861 delTGAG	p.S1286fs	path/ path/ NA/ path/ path	Ser	56	III/ 3	NA
6	S	BRCA2	c.8488-1G>A	p.Œ_splice	path/ NA/ NA/ VUS/ path	Ser	56	III /3	NA
7	G	BRCA2	c.2339C>G	p.S780*	NA/ NA/ path/ NA/ NA	Ser	71	II/ 3	No
8	G	BRCA2	c.773_774 delAA	p.E260fs	NA/ NA/ NA/ NA/ NA	Ser	69	II/ 3	NA
9	S	BRCA1	c.726delT	p.S242fs	NA/ NA/ NA/ NA/ NA	Ser	78	III/ 3	NA
10	S	BRCA1	c.2964delA	p.F989fs	NA/ NA/ NA/ NA/ NA	Ser	63	III/ 3	No
11	S	BRCA1	c.5211_5212 delAG	p.G1738fs	NA/ NA/ NA/ NA/ NA	Ser	49	III/ 3	No
12	S	BRCA2	c.4351delG	p.D1451fs	NA/ NA/ NA/ NA/ NA	Ser	65	III/ 3	No

#*HGVSp* - the Human Genome Variation Society (HGVS) protein sequence name. The Annotation is based on the BRCA1 transcript ENSG00000012048 (NM_007294) and the BRCA2 transcriptENSG00000139618 (NM_000059). §Histological subtype; ser = Serous. ¶ Family history (FH) refers to a positive history of breast and/or ovarian cancer in the first- and second-degree relatives.

Cases in bold letter are novel mutations.

Databases: BIC (Breast Cancer Information Core, http://research.nhgri.nih.gov/bic/), ClinVar (http://www.ncbi.nlm.nih.gov/clinvar/) database, LOVD (Leiden Open Variation Database, http://www.lovd.nl/3.0/home), ARUP (http://arup.utah.edu/database/BRCA/) database and BRCA Share (http://www.umd.be/BRCA1/) database

Abbreviations: NA, not applicable; path, pathogenic; VUS, variant of uncertain significance; y, years.

### Patient characteristics and distribution of *BRCA1/2* mutations in this cohort

**Figure 1:**
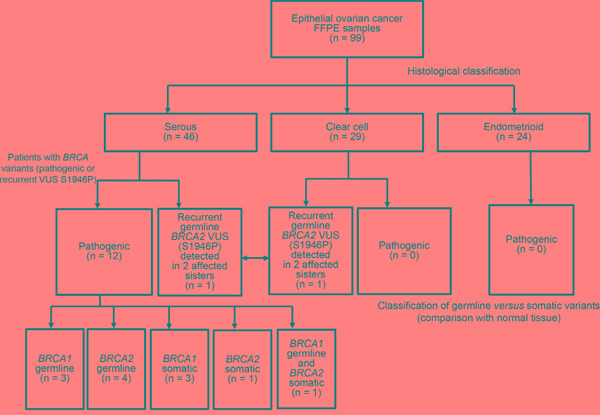
BRCA1/2 genetic variants identified in the study cohort Distribution of pathogenic *BRCA1/2* mutations according to different histological subtypes. The recurrent *BRCA2* VUS p.S1946P was deemed to be pathogenic owing to its occurrence in two sisters with ovarian cancer (one with clear cell carcinoma and the other with serous carcinoma).

The distribution of pathogenic and potentially pathogenic *BRCA1* and *BRCA2* variants according to tumor histological subtypes is depicted in Figure 1. Table 2 summarizes the patient characteristics according to the *BRCA* mutation status.

**Table 2:** Clinical characteristics of the study patients according to the *BRCA* mutation status

		Entire cohort	*BRCA*-positive patients	*BRCA*-negative patients	P
		**n (%)**	**n (%)**	**n (%)**	
		99 (100)	12 (12.1)	87 (87.9)	
Histology					0.001
	Serous carcinoma	46 (46.5)	12 (26.1)	34 (73.9)	
	Endometrioid carcinoma	24 (24.2)	0 (0)	24 (100)	
	Clear cell carcinoma	29 (29.3)	0 (0)	29 (100)	
Age, years					0.069
	Median	52	56	52	
	Range	23−83	42−78	23−83	
	Mean ± SD	52.9 ± 11.1	58.4 ± 10.8	52.2 ± 11.0	
FIGO stage					0.228
	I, II	42 (42.4)*	3 (7.1)#	39 (92.9)#	
	III, IV	57 (57.6)*	9 (15.8)§	48 (84.2)§	
Grade※					0.579
	1	5 (7.1)¶	0 (0) ǂ	5 (100) ǂ	
	2, 3	65 (92.9)¶	12 (18.5)ψ	53 (81.5)ψ	

Patients with pathogenic *BRCA1/2* mutations were considered as *BRCA*-positive, whereas the remaining patients were regarded as *BRCA*-negative. Comparisons between *BRCA*-positive and *BRCA*-negative patients were performed with Fisher's exact tests, χ^2^tests, or Student's *t*-tests, as appropriate.

※Clear cell carcinomas were not graded.

*Percentage calculated on the entire cohort (n = 99).

#Percentage calculated on patients with FIGO stages I and II (n = 42).

§ Percentage calculated on patients with FIGO stages III and IV (n = 57).

¶Percentage calculated on patients whose tumors were graded (n = 70).

ǂPercentage calculated on patients with grade 1 tumors (n = 5).

ψPercentage calculated on patients with grade 2–3 tumors (n = 65).

Twelve patients carried pathogenic *BRCA1/2* mutations. Specifically, *BRCA1/2* germline mutations were identified in seven patients (*BRCA1*, n = 3; *BRCA2*, n = 4), *BRCA1/2* somatic mutations in four patients (*BRCA1*, n = 3; *BRCA2,* n = 1), whereas one patient carried both a *BRCA1* germline mutation and a *BRCA2* somatic mutation. All of the 12 *BRCA*-positive patients had a diagnosis of serous ovarian carcinomas (P = 0.001). The frequencies of *BRCA1/2* pathogenic mutations in the entire cohort and the subgroup of patients with serous carcinoma were 12.1% (12/99) and 26.1% (12/46), respectively. Somatic and germline *BRCA1/2* mutation rates in serous ovarian cancer are 4/46 (8.7%) and 8/46 (17%), respectively. The mean age at onset for patients with and without pathogenic *BRCA1/2* variants was 56 years and 52 years, respectively (P = 0.069). Carriers of *BRCA* germline mutations tended to have a lower median age at diagnosis (55 years) compared with women bearing only somatic mutations (64 years). In the entire study cohort, 57.6% of patients (n = 57) had advanced disease (FIGO stage III–IV). Moreover, 92.9% of patients (n = 65) with serous or endometrioid carcinomas had high-grade malignancies. Of them, 12 patients harbored pathogenic *BRCA1/2* variants. Tumor grade is not routinely determined in clear cell carcinoma [32]. Notably, we observed a recurrent *BRCA2* variant of uncertain significance (VUS) in two sisters (one with clear cell carcinoma and the other with serous carcinoma; Figure 1).

### Pathogenic variants in *BRCA1* and *BRCA2*

**Figure 2:**
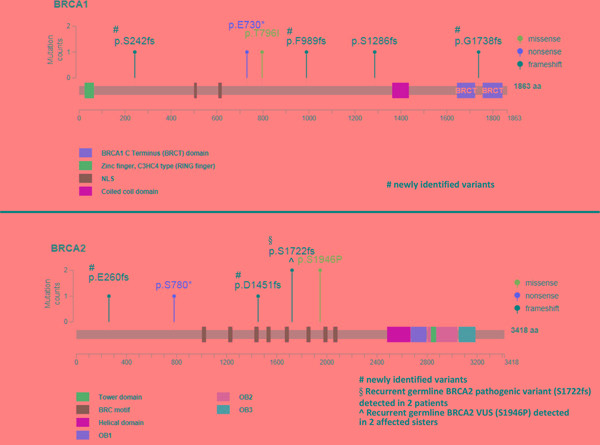
Pathogenic BRCA1/2 variants identified in the study cohort according to their amino acid position The recurrent *BRCA2* VUS p.S1946P—deemed to be pathogenic owing to its occurrence in two sisters with ovarian cancer—is included. However, splice site mutations are not displayed.

Thirteen pathogenic *BRCA1/2* variants were identified in 12 patients (Table 1). Two patients carried the same variant (*BRCA2* p.S1722fs), whereas one patient (subject 6) had two pathogenic variants (one in *BRCA1* and one in *BRCA2*). Of the 13 pathogenic variants, eight were frameshift mutations, two nonsense mutations, one missense mutation, and two splice donor variants. Seven mutations were detected in *BRCA1* and five in *BRCA2*. Five novel frameshift mutations were identified, including four somatic variants (*BRCA1* p.S242fs, *BRCA1* p.F989fs, *BRCA1* p.G1738fs, and *BRCA2* p.D1451fs) and a germline variant (*BRCA2* p.E260fs; Figure 2 and Supplementary Figure S2). There were seven pathogenic *BRCA1/2* germline mutations and five pathogenic somatic mutations (Figure 2).

### VUS and benign variants in *BRCA1* and *BRCA2*

Of the 99 patients, 19.2 % (n = 19) had *BRCA1/2* VUS, all of them being missense variants (Supplementary Table S1). The histological subtypes in the 19 patients with *BRCA1/2* VUS included serous (n = 10; 52.6%), endometrial (n = 5; 26.3%), and clear cell (n = 4; 21.2%) carcinomas. One patient with serous ovarian cancer (subject 7) carried an additional pathogenic variant. A total of 23 VUS were detected, with two patients harboring two VUS (subject 22 and 26) and one patient harboring three VUS (subject 14). One of the three VUS identified in this patient was also evident in another study participant (subject 13). Fifteen (63.6%) and seven (30.4%) VUS were germline and somatic, respectively. One *BRCA2* variant was not classifiable because DNA from non-tumor control tissues was not available. Four benign variants were identified in eight patients, i.e., *BRCA1* variants p.V1247I and p.M1628T, *BRCA2* variant p.I1929V (identified in four patients), and p.R2842H (identified in two patients).

Nine of the detected VUS have not been previously described. Of note, six novel VUS (three in BRCA1 and three in BRCA2) are predicted to be pathogenic by at least one of Grantham/ SIFT/ Polyphen and not present in 997 healthy volunteers (Supplementary Table S1), suggesting their pathogenic potential.

### Identification of a potentially pathogenic *BRCA* VUS

**Figure 3:**
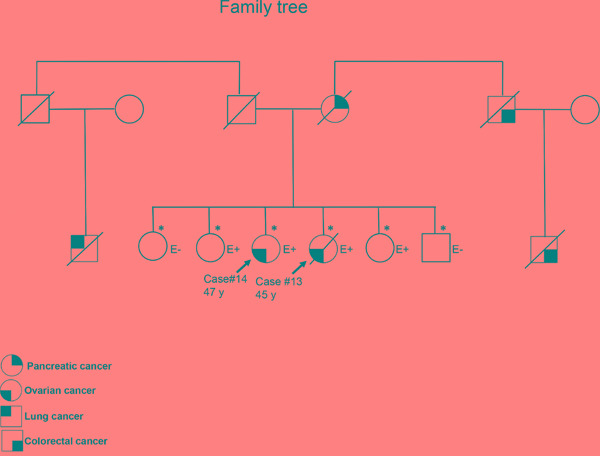
Family tree of the two sisters with ovarian cancer harboring the BRCA2 VUS p.S1946P The asterisks denote subjects who have been tested for the *BRCA2* VUS p.S1946P. Subjects with and without the variant of interest are reported as E+ and E-, respectively.

Three patients carrying a VUS had a family history of breast or ovarian cancer in the absence of any additional pathogenic *BRCA* variant. One patient (subject 24) carried a variant predicted to be pathogenic according to the Grantham and SIFT scores but classified as benign by PolyPhen. The remaining two patients were two sisters (one with clear cell carcinoma and the other with serous carcinoma) who carried the *BRCA2* VUS p.S1946P (predicted to be pathogenic by Grantham but classified as benign by SIFT and PolyPhen). The sister with serous carcinoma carried a *BRCA2* LOH (Table 3), consistent with the two-hit hypothesis of tumor suppressor gene inactivation. The variant is rare in healthy subjects of the Taiwanese population (Supplementary Table S1). To shed more light on the potential pathogenic role of the *BRCA2* VUS p.S1946P, the family history of cancer was analyzed (Figure 3). Among the patients’ four siblings, no cases of cancer were evident. The analysis of germline variants in the patient siblings revealed that two of the patients’ sisters were *BRCA2* p.S1946P carriers, whereas the other sister and the brother were not. From the paternal side, the only known case of malignancy was a lung cancer in a paternal cousin. However, the patients’ mother had pancreatic cancer and their maternal uncle (as well as his son) had colorectal cancer. Although hereditary cancer was plausible, no samples were available from the three family members to investigate the presence of the *BRCA2* VUS p.S1946P. However, the tumor samples of the two ovarian cancer patients were analyzed for the presence of pathogenic variants in the coding regions of the 29 genes involved in DNA repair (*ATM, ATR, BLM, BRIP1, CHEK1, CHEK2, ERCC1, FANCA, FANCC, FANCD2, FANCF, MLH1, MRE11A, MSH2, MSH6, PALB2, PER1, PMS1, PMS2, PRKDC, PTEN, RAD50, RECQL4, SMUG1, TP53, WRN, XPA, XPC,* and *XRCC2)*. However, no shared pathogenic variants were evident (data not shown). Notably, the *BRCA2* VUS p.S1946P is located nearby the BRC motif (Figure 2) which is essential for BRCA2-mediated recombination repair. We thus speculate that the serine-to-proline amino substitution may potentially exert pathogenic effects via an altered BRC conformation.

**Table 3:** Clinical feasibility of PARP inhibitors in patients harboring pathogenic *BRCA1/2* mutations and in two sisters carrying the *BRCA2* VUS p.S1946P

ID	Variant classification	Gene	Amino acid change#	Variant frequency (%)	Germline (G)/ somatic (S)	LOH/ biallelic inactivation	Eligibility for PARPi (USFDA label)	Eligibility for PARPi (EMA label)
1	pathogenic	BRCA2	p.S1722fs	78	G	Yes	Yes	Yes
2	pathogenic	BRCA2	p.S1722fs	74	G	Yes	Yes	Yes
3	pathogenic	BRCA1	p.E730*	77	G	Yes	Yes	Yes
4ψ	pathogenic	BRCA1	p.T796I	53	G	Unknown§	Yes	Yes
5	pathogenic	BRCA1	p.Œ_splice	78	G	Yes	Yes	Yes
6	pathogenic	BRCA1	p.S1286fs	62	G	Yes	Yes	Yes
7	pathogenic	BRCA2	p.S780*	80	G	Yes	Yes	Yes
8	pathogenic	BRCA2	p.E260fs	75	G	Yes	Yes	Yes
9	pathogenic	BRCA1	p.S242fs	79	S	Yes	No	Yes
10	pathogenic	BRCA1	p.F989fs	59	S	Yes	No	Yes
11	pathogenic	BRCA1	p.G1738fs	68	S	Yes	No	Yes
12	pathogenic	BRCA2	p.D1451fs	43	S	Yes	No	Yes
13	VUS	BRCA2	p.S1946P	75	G	Yes	Yes¶	Yes¶
14	VUS	BRCA2	p.S1946P	48	G	No	Yes¶	Yes¶
**Patients eligible for PARPi (Yes/Unknown/No)**							**10/0/4**	**14/0/0**

ψPatient 4 harbored an additional somatic pathogenic BRCA2 variant characterized by a low frequency (6%). #*HGVSp* - the Human Genome Variation Society (HGVS) protein sequence name. The Annotation is based on the BRCA1 transcript ENSG00000012048 (NM_007294) and the BRCA2 transcript ENSG00000139618 (NM_000059). §The SNP analysis did not allow a conclusion about the presence of an LOH. ¶Based on the assumption that the VUS p.S1946P can be classified as pathogenic.

Abbreviations: EMA, European Medicines Agency; LOH, loss of heterozygosity;PARPi, poly (ADP-ribose) polymerase inhibitors; USFDA, United States Food and Drug Administration; VUS, variant of uncertain significance.

### Biallelic *BRCA1/2* inactivation and its therapeutic implications

We hypothesized that the presence of a biallelic *BRCA* inactivation (regardless of the germline or somatic origin of the investigated variant) may identify patients who benefit from PARPi. The presence of LOH was tested in 14 patients, of whom 12 had pathogenic *BRCA* mutations (Table 3) and the remaining two were the sisters harboring the *BRCA2* germline variant p.S1946P. One patient carried both a germline and a somatic *BRCA* mutation (subject 6), but only the germline mutation was tested. A biallelic inactivation of the mutated *BRCA* gene by LOH was detected in 12 patients (85.7%). Importantly, LOH was identified in all of the four patients who carried somatic pathogenic *BRCA* mutations. Of the 10 patients with germline *BRCA* mutations, eight had a LOH, whereas no LOH could be detected in two patients. However, in one case the tumor purity was very low, which is insufficient for LOH detection. According to the US FDA, the use of PARPi in our patients would be limited to cases with *BRCA* germline mutations (n = 10), whereas all patients were eligible for PARPi under the EMA label.

## DISCUSSION

Herein we reported five novel pathogenic *BRCA1/2* mutations (Table 1) and six novel VUS with pathogenic potential (Supplementary Table S1) in patients with ovarian cancer. These results expand our knowledge on the occurrence of both germline and somatic *BRCA1/2* mutations in Taiwanese patients with different histological subtypes of ovarian cancer. Screening of *BRCA* mutations in patients with ovarian cancer may have implications for allocating patients to PARPi.

We also confirmed the findings of a recent report regarding the feasibility of using FFPE tumor samples for NGS analysis of alterations in the *BRCA1/2* genes [29]. To our knowledge, only one *BRCA1* germline stop mutation has been previously identified by single-strand conformation polymorphism in a sample of 68 Taiwanese patients with ovarian cancer [21]. By using NGS, we were able to identify deleterious *BRCA1/2* mutations in 12.1% of our patients, with germline mutations being evident in 8.1% of the samples. A similar prevalence of germline mutations has been reported in Chinese patients (6.9%) [33], whereas a higher frequency has been observed in Japanese (12.6%) [28] and Western cohorts (13.2−15.3%) [8, 34, 35]. A Korean study found *BRCA1/2* mutations in 13.5% of patients who had a negative family history [36]. The prevalence of pathogenic germline mutations identified in our patients with serous tumors (17.4%) was in accordance with that of Japanese (16.2%) [28].

The combined prevalence of *BRCA1/2* germline and somatic mutations in patients with ovarian cancer differs according to the detection method used (Table 4). However, the reported frequency of *BRCA1/2* mutations in patients with high-grade serous carcinoma has been shown to vary between 19 and 30% [5, 6, 27, 29], which is in line with the results of our study (26.1%). Similarly, the observed frequency of somatic mutations in our cohort (33%) is in line with the published literature (16−40%) [5, 6, 27, 29, 37]. The *BRCA* mutation rate in endometrioid carcinoma of the ovary is ≤ 10% [8, 28, 33, 34]. Albeit less investigated, a similarly low frequency has been reported for clear cell carcinomas [25, 26, 28, 34], a feature that may explain their generally poor response to platinum-based chemotherapy [38]. Here, we were unable to identify pathogenic *BRCA1/2* mutations in patients with endometrioid or clear cell carcinomas. However, we observed a potentially deleterious *BRCA2* germline variant (*BRCA2* p.S1946P) in two sisters with ovarian cancer (one with clear cell carcinoma and the other with serous carcinoma). The patients had a positive family history of malignancies (including pancreatic and colorectal cancer) known to be associated with *BRCA* mutations [39, 40]. We initially reasoned that the malignancies of the two patients could have been caused by the Lynch syndrome [41] or mutations in *TP53* or other DNA repair genes [42–44]. However, both sisters did not show any shared pathogenic variant in 29 DNA repair genes (including *TP53, MLH1, MSH2, MSH6*, *PMS2* and genes involved in homologous recombination). Moreover, the change from serine to proline may affect protein conformational changes locally or globally and change protein functions [45]. pS1946P is located in exon 11 where the BRC motif binds to RAD51 and belongs to the ovarian cancer cluster region (OCCR). Mutations in this region have been shown to confer a higher risk for ovarian cancer [46, 47]. Taken together, our data suggest that the VUS *BRCA2* p.S1946P may be pathogenic.

**Table 4:** Prevalence of germline and somatic *BRCA1/2* mutations in ovarian cancer

Authors	Samples	Somatic plus germline % (n/N)	Somatic only % (n/N)	Analytical method
**High-grade serous ovarian carcinomas**				
Mafficini, 2016 [[Bibr R29]]	FFPE	28 (13/47)	23 (3/13)	NGS (Ion Torrent)
McAlpine, 2012 [[Bibr R27]]	Fresh	30 (31/103)	16 (5/31)	Illumina exome sequencing, DHPLC (part), MLPA
TCGA, 2011 [[Bibr R5]]	Fresh	22 (70/316)	27 (19/70)	NGS
Hennessy, 2010 [[Bibr R6]]	Fresh	19 (44/235)	39 (11/28)	Agilent high-density tiling array
Current study	FFPE	26 (12/46)	33 (4/12)	NGS (Ion Torrent)
**High-grade serous and non-serous ovarian carcinomas**				
Hilton, 2002 [[Bibr R37]]	Fresh	33 (30/92)	40 (12/30)	PTT
Current study	FFPE	12 (12/99)	33 (4/12)	NGS (Ion Torrent)

Abbreviations: FFPE, formalin-fixed paraffin-embedded; DHPLC, denaturing high-performance liquid chromatography; MLPA, multiplex ligation-dependent probe amplification; NGS, next-generation sequencing; PTT, protein truncation test; TCGA, The Cancer Genome Atlas Research Network.

We then analyzed the eligibility for PARPi of the 14 patients who carried either pathogenic *BRCA1/2* mutations or the *BRCA2* VUS p.S1946P. Only 10 patients (71.4%) met the FDA criteria for use of PARPi (owing to the presence of germline mutations), whereas all of them should have been eligible according to the EMA guidelines. Biallelic *BRCA1/2* inactivation was also considered, because tumor cells with monoallelic *BRCA* inactivation are unresponsive to PARPi [20]. In our study, *BRCA1/2* inactivation was identified in most – but not all – patients with pathogenic or potentially deleterious *BRCA* variants. However, an accurate estimation of the proportion of patients carrying monoallelic *BRCA1/2* inactivation was unfeasible because of the small sample size.

There were several limitations in our study. First, we did not analyze whether *BRCA1/2* genes were inactivated by epigenetic silencing [5]. Second, other genes associated with homologous recombination deficiency in patients with ovarian cancer patients were not analyzed [5, 10]. Consequently, we cannot exclude that the proportion of patients eligible for treatment with PARP inhibitors may have been underestimated. Third, the sample size of this study is small especially in the group of serous carcinoma. However, we provide the mutation rates in the understudied East Asian region, where we showed similar mutation rates to those reported in Caucasians and other ethnic subgroups. Fourth, the family history of each studied case was only retrieved from charts. Of note, none of the germline *BRCA1/2* had a family history of breast and ovarian cancer. Careful cancer screening on the family members of any detected pathogenic germline *BRCA1/2* mutation should be advised.

In conclusion, *BRCA1/2* mutations are common in Taiwanese patients with serous ovarian carcinoma. Several novel *BRCA1/2* mutations reported herein warrant further validation for their pathogenic potentials. The identification of *BRCA1/2* somatic mutations may have implications for guiding therapeutic decisions in patients with ovarian cancer.

## MATERIALS AND METHODS

### Patients

The study protocol was approved by the Institutional Review Board of the Chang Gung Memorial Hospital (IRB No. 104-6241B). Ninety-nine patients with ovarian cancer were included. The histological variants included serous (n = 46), endometrioid (n = 24), and clear cell (n = 29) carcinomas. The occurrence of breast and ovarian cancer in first- and second-degree relatives was investigated.

### Samples and DNA extraction

We used FFPE samples obtained by surgical removal of the primary tumors. Normal FFPE samples were used as paired controls for specimens carrying genetic variants. Genomic DNA was isolated from two 10 μm-thick FFPE sections using the QIAamp DNA FFPE Tissue Kit (Qiagen, Hilden, Germany). We obtained blood samples from the family members of the two sisters harboring the *BRCA2* VUS p.S1946P. Genomic DNA was extracted from blood white cells using the QIAamp DNA Blood Midi Kit (Qiagen). The concentration and integrity of the purified DNA were checked using the Quanti-iT dsDNA HS Assay (Invitrogen, Carlsbad, CA, USA) and a Fragment Analyzer (Advanced Analytical Technologies, Ankeny, IA, USA), respectively.

### *BRCA1/2* sequencing and data processing

Genomic DNA (40 ng) was amplified using 250 primer pairs (GeneRead DNASeq Targeted panels v2, Qiagen) to target all of the exonic regions as well as the intronic regions within 20-bp of a splicing junction. Amplicons were ligated to a barcode adaptor using the Ion Xpress™ Plus Fragment Library Kit (Life Technologies, Carlsbad, CA, USA). The barcoded library was then enriched by emulsion PCR using OneTouch2 and OneTouch ES instruments (Life Technologies) following the Ion Torrent protocol provided by Life Technologies. The library's quantity and quality were examined on a fragment analyzer (Advanced Analytical Technologies) and a Qubit fluorometer (Invitrogen). The enriched library was sequenced using the Ion Personal Genome Machine (PGM) with an Ion 318 chip (Life Technologies) following the manufacturer's instructions. The mean sequencing depth for FFPE tumor samples was >5700×, with a mean uniformity of 91.1%. Variants with a frequency >10% were confirmed by Sanger sequencing of tumor DNA. Germline DNA was analyzed by NGS or Sanger sequencing. Germline DNA from the siblings of the two sisters carrying the *BRCA2* VUS p.S1946P was also examined by Sanger sequencing.

### Analysis of genetic variants

Raw sequence data were mapped to the human reference genome (hg19) using The Torrent Suite Server (v. 4.2). Variant calling was performed with the Torrent Suite Variant Caller plug-in (v. 4.2) and the Variant Effect Predictor (VEP) was used for annotation. Variants with a read count <25 and a variant frequency <5% were not analyzed further. Common single nucleotide polymorphisms (SNPs) were identified using the dbSNP (release 138), 1000 Genome (phase 1 data), and 5000 Exome data sets. Previously reported *BRCA1/2* mutations were identified and classified with the BIC (Breast Cancer Information Core, http://research.nhgri.nih.gov/bic/), ClinVar (http://www.ncbi.nlm.nih.gov/clinvar/), LOVD (Leiden Open Variation Database, http://www.lovd.nl/3.0/home), ARUP (http://arup.utah.edu/database/BRCA/), and BRCA Share (http://www.umd.be/BRCA1/) data sets. A variant classified as “likely benign” or “likely pathogenic” by the ClinVar was considered as VUS. Variants were classified as pathogenic if 1) they were labeled as such in any of the data sets used for the study or 2) they were frameshift or stop mutations. Variants were considered as benign if they were unequivocally classified as such (i.e., without a concurrent classification either as VUS or pathogenic within the same database) in the consulted data sets. All of the previously unidentified variants that were not clearly benign or pathogenic were regarded as VUS. A pathogenic variant was considered as novel if it was absent in the abovementioned data sets as well as in the COSMIC database (version 70, http://cancer.sanger.ac.uk/cosmic). SIFT (http://sift.jcvi.org/), PolyPhen2 (http://genetics.bwh.harvard.edu/pph2/index.shtml), and Grantham (http://asia.ensembl.org/info/genome/variation/predicted_data.html) were used to predict the functional impact of the detected variants (Supplementary Table S2). Variants classified as “probably damaging” in PolyPhen2 were considered as pathogenic, whereas “possibly damaging” variants were classified as VUS. SNP data of 997 healthy subjects of the population-based project in Taiwan were downloaded for comparison (https://taiwanview.twbiobank.org.tw/index).

In line with previous methodology [48], LOH was determined by analyzing the frequency of the patient's SNPs within the mutated *BRCA* gene using the ADTEx tool. This method may also detect large genomic rearrangements.

### Statistical analysis

Intergroup differences were analyzed using Fisher's exact tests, χ^2^ tests, or Student's *t*-tests, as appropriate. All calculations were performed using the IBM SPSS software package (version 17.0; IBM, Armonk, NY, USA). P values < 0.05 (two-tailed) were considered statistically significant.

## SUPPLEMENTARY MATERIALS FIGURES AND TABLES


