# Supplementary material for: Prevalence and clinical significance of BRCA1/2 germline and somatic mutations in Taiwanese patients with ovarian cancer
Source: Oncotarget. 2016 Nov 19;7(51):85529–41. doi: 10.18632/oncotarget.13456 (PMC5356755; doi:10.18632/oncotarget.13456)
Supplement: Supplementary file 1 [file oncotarget-07-85529-s001.pdf]

Prevalence and clinical significance of *BRCA1/2* germline and somatic mutations in Taiwanese patients with ovarian cancer

SUPPLEMENTARY FIGURES AND TABLES

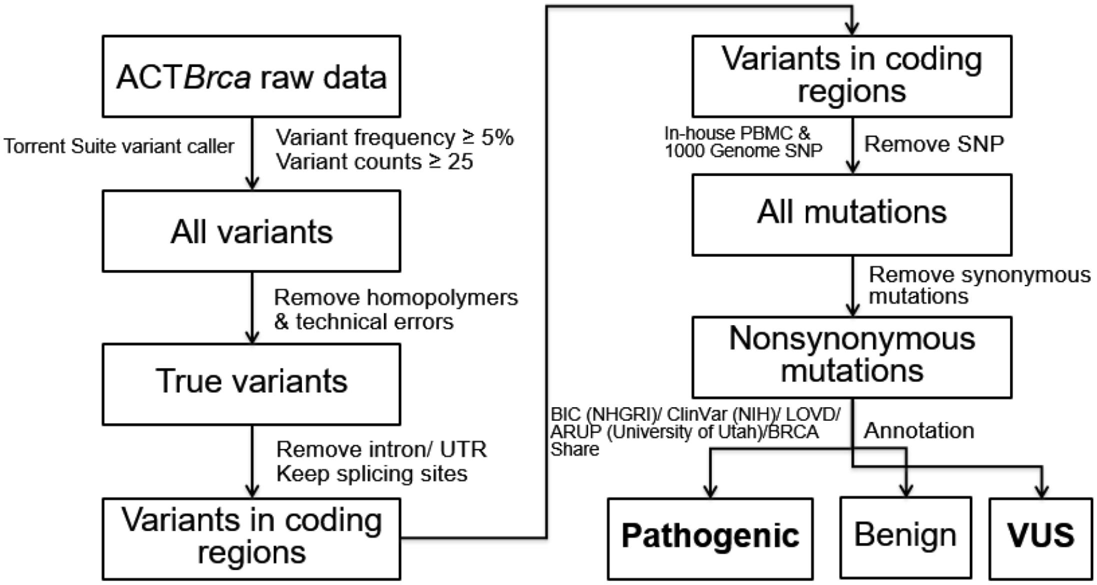

Supplementary Figure S1: Workflow of identification and annotation of BRCA1/2 variants.
Databases: BIC (Breast Cancer Information Core, http://research.nhgri.nih.gov/bic/), ClinVar (http://www.ncbi.nlm.nih.gov/clinvar/) database, LOVD (Leiden Open Variation Database, http://www.lovd.nl/3.0/home), ARUP (http://arup.utah.edu/database/BRCA/) database and BRCA Share (http://umd.be/brca1) database
Abbreviations: PBMC, peripheral blood mononuclear cell; SNP, single nucleotide polymorphism; UTR, untranslated region; VUS, variant of uncertain significance.

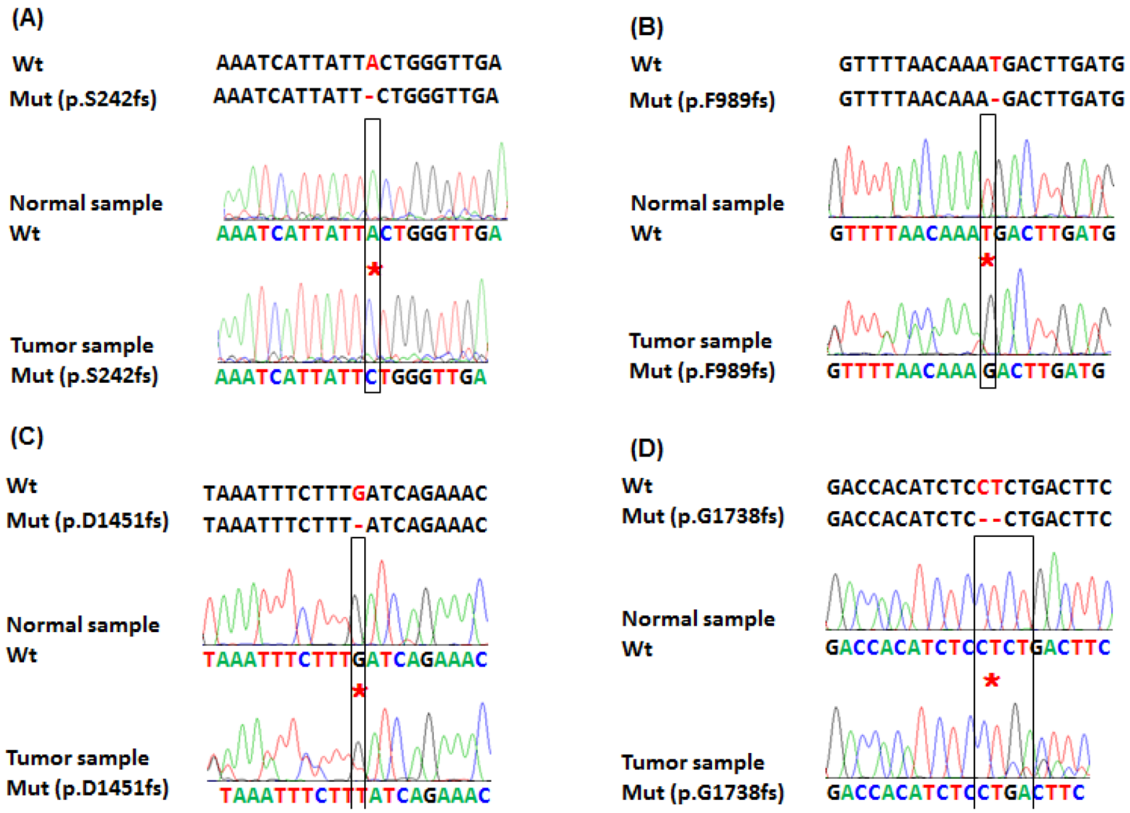

Supplementary Figure S2: Validation of novel pathogenic mutations by Sanger sequencing. Sanger sequencing was performed to validate mutations detected by next generation sequencing. Plots are shown for the four novel somatic mutations. The absence of mutations in germline DNA reveals the somatic origin of the mutations.

Supplementary Table S1: Description of *BRCA* variants and clinical characteristics of patients carrying VUS.

ID	Germline (G)/ Somatic (S)	Gene	Change for nucleotides (nt) and amino acids (aa)#		Previously reportedψ	Predicted impact (Grantham/ SIFT/ PolyPhen)	Type §	Age (y)	FIGO stage/ grade ǂ	FH¶	Population frequency in TaiwanViewζ (n = 997)
			nt	aa							
Recurrent variants (n = 4)											
13	G	BRCA2	c.5836T>C	p.S1946P	Yes	path/ ben/ ben	Ser	45	II/ 2	Yes	0.1% (C/T) 99.9% (T/T)
14	G	BRCA2	c.5836T>C	p.S1946P	Yes	path/ ben/ ben	CC	47	I/ NA	Yes	0.1% (C/T) 99.9% (T/T)
15	G	BRCA1	c.1036C>T	p.P346S	Yes	path/ ben/ ben	CC	55	I NA	No	0.8% (A/G) 99.2% (G/G)
16	G	BRCA1	c.1036C>T	p.P346S	Yes	path/ ben/ ben	Ser	45	I/ 3	NA	0.8% (A/G) 99.2% (G/G)
Unique variants (n = 19)											
7	G	BRCA1	c.2969T>C	p.V990A	**No**	path/ ben/ ben	Ser	71	II/ 3	No	NA
17	G	BRCA1	c.811G>A	p.V271M	Yes	ben/ ben/ unknown	Ser	73	III/ 2	NA	0.1% (C/T) 99.9% (C/C)
18	G	BRCA1	c.4748G>A	p.R1583K	Yes	ben/ ben/ ben	En	42	I/ 2	NA	0.1% (C/T) 99.9% (C/C)
19	G	BRCA2	c.7522G>A	p.G2508S	Yes	ben/ path/ path	Ser	67	III/ 3	NA	0.3% (A/G) 99.7% (G/G)
20	G	BRCA2	c.7052C>G	p.A2351G	Yes	path/ ben/ unknown	Ser	65	III/ 3	NA	0.4% (C/G) 99.6% (C/C)
21	G	BRCA2	c.4376A>G	p.N1459S	Yes	ben/ ben/ ben	CC	57	II/ NA	NA	NA
22	G	BRCA2	c.6325G>A	p.V2109I	Yes	ben/ ben/ ben	Ser	44	III/ 3	NA	1.1 % (A/G) 98.9% (G/G)
22	G	BRCA2	c.9875C>T	p.P3292L	Yes	path/ path/ path	Ser	44	III/ 3	NA	0.2% (C/T) 99.8% (C/C)
23	G	BRCA2	c.943T>A	p.C315S	Yes	path/ ben/ ben	En	52	I/ 2	NA	0.8 (A/T) 99.2% (T/T)
24	G	BRCA2	c.5231G>T	p.S1744I	**No**	path/ path/ ben	CC	51	III/ NA	Yes	0.1% (G/T) 99.9% (G/G)
25	G	BRCA2	c.4599A>C	p.K1533N	Yes	path/ ben/ ben	Ser	65	III/3	NA	0.4% (A/C) 99.6% (A/A)
26	S	BRCA1	c.2261G>A	p.G754E	**No**	path/ ben/ ben	Ser	45	III/ 3	NA	NA
26	S	BRCA1	c.5349G>A	p.M1783I	**No**	ben/ path/ ben	Ser	45	III/ 3	NA	NA
27	S	BRCA1	c.4036G>A	p.E1346K	Yes	ben/ ben/ ben	En	54	I/ 2	NA	NA
28	S	BRCA2	c.658G>A	p.V220I	**No**	ben/ ben/ ben	En	42	I/ 2	No	NA
29	S	BRCA2	c.6463C>A	p.L2155I	**No**	ben/ ben/ ben	En	51	I/ 1	No	NA
14	S	BRCA2	c.3716A>C	p.K1239T	**No**	path/ path/ unknown	CC	47	I/ NA	Yes	NA
14	S	BRCA2	c.3722T>G	p.F1241C	**No**	path/ path/ path	CC	47	I/ NA	Yes	NA
30	NA	BRCA2	c.1732G>C	p.G578R	**No**	path/ path/ path	Ser	50	IV/ 3	No	NA

#HGVSp - the Human Genome Variation Society (HGVS) protein sequence name. The Annotation is based on the BRCA1 transcript ENSG00000012048 (NM_007294) and the BRCA2 transcriptENSG00000139618 (NM_000059). ψVariant found in ARUP, BIC, BRCA Share, ClinVar, LOVD or COSMIC datasets. §Histological subtype; Ser = serous, En = endometrioid, CC = clear cell. ǂClear cell carcinomas are not graded. ¶Family history (FH) refers to breast and/or ovarian cancer in first and second degree relatives. ζTaiwan Biobank https://taiwanview.twbiobank.org.tw/index.
Abbreviations: ben, benign; NA, not applicable; path, pathogenic; VUS, variant of uncertain significance; y, years.

Supplementary Table S2: Prediction of pathogenicity of BRCA1/2

See Supplementary File 1
